# Editorial: Clinical Therapeutic Development Against Cancers Resistant to Targeted Therapies

**DOI:** 10.3389/fphar.2021.816896

**Published:** 2022-01-12

**Authors:** Fanfan Zhou, Hong Zhu, Caiyun Fu

**Affiliations:** ^1^ Sydney Pharmacy School, Faculty of Medicine and Health, The University of Sydney, Sydney, NSW, Australia; ^2^ Zhejiang Province Key Laboratory of Anti-Cancer Drug Research, College of Pharmaceutical Sciences, Zhejiang University, Hangzhou, China; ^3^ Zhejiang Provincial Key Laboratory of Silkworm Bioreactor and Biomedicine, College of Life Sciences and Medicine, Zhejiang Sci-Tech University, Hangzhou, China

**Keywords:** cancer resistance, targeted therapies, therapeutic development, anti-cancer therapeutics, therapeutic resistance

## Introduction

Cancer is one of the primary causes of death that can affect any organ of the body. Cancer originates from transforming normal cells to tumour cells that often rapidly propagate and invade into adjoining tissues. Cancer cells spreading into other tissues are so called metastases, which is highly associated with death in humans. As to the World Health Organization, lung, colon and liver cancers are among the top ranked cancer types with high mortalities in recent years. The burden of cancer can be largely reduced with proper prevention, early diagnosis, effective treatment, and appropriate palliative care. Cancer treatment remains challenging, largely due to the complexity of its etiology and frequent occurrence of drug resistance.

Targeted therapies are preferably adopted in cancer treatment for years. Such treatment alone or in combination with conventional chemotherapies have improved the survival of many cancer patients including those with tumours considered as incurable. Although clinical successes have been achieved in many cases, the failure rate of cancer targeted therapies remains disappointingly high. This is possibly due to the misapplication of therapies targeting pan-essential genes, the dysfunction of which leads to dose-limiting toxicities and/or the compromised therapeutic efficacy resulted from drug resistance.

Drug resistance is either early intrinsic or late acquired (Groenendijk and Bernards, 2014). The versatility of tumours to therapies as well as the heterogeneity of patients and tumours contribute to the fast adaptivity of tumours to treatment and evolution of resistant tumour mutations. Literature showed that the close crosstalk among signalling pathways is one of the primary leads to cancer drug resistance, for example that of the ER and HER2 pathways in breast cancer (Gu et al., 2016). Although simultaneously acting on multiple signalling is a preferred approach in cancer treatment, target desensitization and recurrent variants have been widely detected in cancer patients, which significantly hinder the progress of anti-cancer drug development. Therefore, understanding the mechanisms of cancer drug resistance can provide guidance to optimise existing targeted therapies, identify therapeutic targets valuable to the discovery of new and improved agents as well as form the basis of therapeutic advance in cancer treatment. The possible solutions to overcome drug resistance in confronting cancers not sensitive to existing targeted therapies include combinational and/or personalised therapies, novel drug delivery as well as new agents acting on new therapeutic targets.

### Identification of New Anti-Cancer Therapeutic Targets

Drug resistance is the primary obstacle to achieve satisfactory clinical outcomes in carcer targeted therapies as numerous patients are inherently or adaptively resistant to existing regimens. Furthermore, there are deadly cancers without effective systemic therapies at present, like human uveal melanoma (Wang et al., 2021a) and gastroesophageal cancer (Kasper and Schuler, 2014). Identifying novel therapeutic targets in these cancers is urgently required to develop life-saving regimens.

Proteins that play essential roles in cell division, cell cycle progression and/or cell death continue to be valid therapeutic targets in cancer treatment. For instance, histone deacetylases (HDACs) that are enzymes responsible for removing acetyl groups from proteins, are widely involved in cellular processes. Their inhibitors have shown clinical potentials against specific malignancies and are under pre-clinical and clinical evaluations to treat various cancers (Manzotti et al., 2019).

As to gastroesophageal cancer (ie., gastric cancer and cancers of the distal oesophagus and gastroesophageal junction), targeted therapies against EGFR/HER signalling have been extensively evaluated. Despite the reduced side effects compared to that of conventional chemotherapies, a panel of tyrosine kinase inhibitors (TKIs) downregulating EGFR/HER signalling yielded unsatisfactory outcomes in clinical trials. Thus, emerging drug targets like the store-operated Ca2+ entry (SOCE) (Cui et al., 2017), attract the attentions of researchers endeavoured in drug development for this type of cancer. Besides EGFR/HER signalling, other receptor tyrosine kinase (RTK) families like the fibroblast growth factor (FGF) receptors (Kommalapati et al., 2021) and PI3K/AKT/mTOR signalling (Alzahrani, 2019) that are involved in tumorigenesis and cell proliferation, come under the spotlight in cancer drug development. Small molecules modulating these signallings demonstrate potency in treating various types of cancers and are promising therapeutic options for further evaluation.

### Research Advance in Adjuvant Cancer Regimens

Drug resistance pronouncedly compromises the clinical effectiveness of cancer therapies; there are multiple mechanisms contribute to such therapeutic defect. ATP binding cassette (ABC) transporters are important membrane proteins responsible for cellular efflux of substances including many commonly used drugs. Overexpression of ABCs has been widely observed in tumour cells, which notably leads to clinical failure of cancer targeted therapies (Wang et al., 2021b). One of the preferred strategies to overcome this problem is the co-administration of ABC modulators with standard anti-cancer drugs.

In colorectal cancer, irinotecan is clinically used in treating its metastatic disease. It is known that ABCG2/BCRP-mediated drug resistance remarkedly impacts on the pharmacokinetic performance of irinotecan and consequently, limits the clinical applications of this agent (Nielsen et al., 2017). The inevitable development of resistance largely impacts on treatment efficacy and contributes to the high incidence and mortality rate of this disease. It is expected that co-administering agents that can potently sensitise ABCG2 overexpressing colorectal tumours to clinically adopted targeted therapies, would greatly improve the treatment outcome of this top-ranked deadly cancer.

Empirical approaches and *in silico* analysis have both been adopted to design adjuvant cancer regimens. Overexpressed oncogenes and common biological processes like ubiquitination are preferable targets for adjuvant therapies. Computer modelling-facilitated chemical modification has been widely applied in designing combined therapies in addition to traditional chemotherapies such as cisplatin and doxorubicin with severe side effects. The pre-clinical tests regarding these adjuvant regiments are often conducted on malignant cell lines and tumour xenograft models. However, clinical predictions based on these studies may be suboptimal due to the loss of tumour characteristics in immortalised cell lines and expected species differences between human and animals. Therefore, it is highly desired that confirmative evaluation can be performed on tumour tissue-derived primary cell lines, which better preserve tumour characteristics and genetic variations.

One emerging area in cancer drug research is the incorporation of machine learning algorithms with the advance of artificial intelligence (Tanoli et al., 2021). With the rapidly increased quantity and improved quality of cases archived in databases capturing both patient and treatment information, therapeutic response prediction facilitates the optimisation of cancer treatment with reduced drug resistance and enhanced efficacy (Rafique et al., 2021). However, lack of clinically proven pharmacogenomic data remains one of the primary challenges in this area.

### Development of Novel Drug Delivery Route in Cancer Treatment

It is thought that novel drug delivery carriers can greatly improve effectiveness, safety and targetability of agents. Research has widely investigated unconjugated or conjugated liposomes, polymeric micelles, microspheres and nanoparticles in delivering cancer targeted therapies. The advance in drug delivery technology adds credits to existing cancer targeted therapies with enhanced stability and biocompatibility, improved targeting as well as reduced drug resistance (Yao et al., 2020; Raj et al., 2021). The drawbacks of this approach include unwanted penetration into the brain, potential harmful accumulation in human bodies and possible dangers to the environment. Thus, the careful selection and cautious utilisation of novel drug delivery carriers in cancer treatment are highly desired.

Overall, the ongoing battle against drug resistance associated with cancer targeted therapies is a great challenge in biomedical research. Continuous efforts, multidisciplinary collaborations and long-term monitoring will be required to ensure the progress in this field.

## References

[B1] Alzahrani A. S. (2019). PI3K/Akt/mTOR Inhibitors in Cancer: At the Bench and Bedside. Semin. Cancer Biol. 59, 125–132. 10.1016/j.semcancer.2019.07.009 31323288

[B2] Cui C. Merritt R. Fu L. Pan Z. (2017). Targeting Calcium Signaling in Cancer Therapy. Acta Pharm. Sin B 7, 3–17. 10.1016/j.apsb.2016.11.001 28119804 PMC5237760

[B3] Groenendijk F. H. Bernards R. (2014). Drug Resistance to Targeted Therapies: Déjà Vu All over Again. Mol. Oncol. 8, 1067–1083. 10.1016/j.molonc.2014.05.004 24910388 PMC5528618

[B4] Gu G. Dustin D. Fuqua S. A. (2016). Targeted Therapy for Breast Cancer and Molecular Mechanisms of Resistance to Treatment. Curr. Opin. Pharmacol. 31, 97–103. 10.1016/j.coph.2016.11.005 27883943

[B5] Kasper S. Schuler M. (2014). Targeted Therapies in Gastroesophageal Cancer. Eur. J. Cancer 50, 1247–1258. 10.1016/j.ejca.2014.01.009 24495747

[B6] Kommalapati A. Tella S. H. Borad M. Javle M. Mahipal A. (2021). FGFR Inhibitors in Oncology: Insight on the Management of Toxicities in Clinical Practice. Cancers (Basel) 13, 968. 10.3390/cancers13122968 34199304 PMC8231807

[B7] Manzotti G. Ciarrocchi A. Sancisi V. (2019). Inhibition of BET Proteins and Histone Deacetylase (HDACs): Crossing Roads in Cancer Therapy. Cancers (Basel) 11, 304. 10.3390/cancers11030304 PMC6468908 30841549

[B8] Nielsen D. L. Palshof J. A. Brünner N. Stenvang J. Viuff B. M. (2017). Implications of ABCG2 Expression on Irinotecan Treatment of Colorectal Cancer Patients: A Review. Int. J. Mol. Sci. 18, 1926. 10.3390/ijms18091926 PMC5618575 28880238

[B9] Rafique R. Islam S. M. R. Kazi J. U. (2021). Machine Learning in the Prediction of Cancer Therapy. Comput. Struct. Biotechnol. J. 19, 4003–4017. 10.1016/j.csbj.2021.07.003 34377366 PMC8321893

[B10] Raj S. Khurana S. Choudhari R. Kesari K. K. Kamal M. A. Garg N. (2021). Specific Targeting Cancer Cells with Nanoparticles and Drug Delivery in Cancer Therapy. Semin. Cancer Biol. 69, 166–177. 10.1016/j.semcancer.2019.11.002 31715247

[B11] Tanoli Z. Vähä-Koskela M. Aittokallio T. (2021). Artificial Intelligence, Machine Learning, and Drug Repurposing in Cancer. Expert Opin. Drug Discov. 16, 977–989. 10.1080/17460441.2021.1883585 33543671

[B12] Wang J. Q. Wu Z. X. Yang Y. Teng Q. X. Li Y. D. Lei Z. N. (2021). ATP-binding Cassette (ABC) Transporters in Cancer: A Review of Recent Updates. J. Evid. Based Med. 14, 232–256. 10.1111/jebm.12434 34388310

[B13] Wang J. Z. Lin V. Toumi E. Wang K. Zhu H. Conway R. M. (2021). Development of New Therapeutic Options for the Treatment of Uveal Melanoma. FEBS J. 188, 6226–6249. 10.1111/febs.15869 33838075

[B14] Yao Y. Zhou Y. Liu L. Xu Y. Chen Q. Wang Y. (2020). Nanoparticle-Based Drug Delivery in Cancer Therapy and its Role in Overcoming Drug Resistance. Front. Mol. Biosci. 7, 193. 10.3389/fmolb.2020.00193 32974385 PMC7468194

